# Hedgehog/GLI1 signaling pathway regulates the resistance to cisplatin in human osteosarcoma

**DOI:** 10.7150/jca.61591

**Published:** 2021-09-21

**Authors:** Daosen Chen, Xiaodiao Kang, Zhenxing Li, Liang Chen, Qiong Ma, Pei Fan

**Affiliations:** 1 Department of Orthopedics, The Second Affiliated Hospital of Wenzhou Medical University, Yuying Children's Hospital, Wenzhou 325027, China.; 2 Zhejiang Provincial Key Laboratory of Orthopedics, The Second Affiliated Hospital of Wenzhou Medical University, Yuying Children's Hospital, Wenzhou 325027, China.; 3 Orthopedic Oncology Institute, Department of Orthopedic Surgery, Tangdu Hospital, Fourth Military Medical University, Xi'an 710038, China.

**Keywords:** Hedgehog pathway, GLI1, osteosarcoma, cisplatin resistance, γ-H2AX

## Abstract

**Purpose:** This study aimed to investigate the role and mechanism of Hedgehog/GLI1 signaling pathway in regulating the resistance to cisplatin in osteosarcoma (OS).

**Materials and methods:** Immunohistochemistry, western blotting and qRT-PCR assay were performed to analyze and compare the expression of GLI1 in OS tumor tissue and normal bone tissue as well as in cisplatin sensitive and resistant cell lines (SOSP-9607 and SOSP-9607/CR). Meanwhile, the biological role of GLI1 in OS was investigated by using down-regulated expression of GLI1 and functional assays, including CCK-8, colony formation assay, flow cytometry, and wound healing assay. Moreover, the relationship between GLI1 and γ-H2AX (DNA damage protein) in cells treated with GLI1 siRNA and cisplatin was examined using western blot analysis. In addition, GANT61, a inhibitor of Hedgehog pathway was used in xenograft tumor model to further verify the effect and mechanism of GLI1 on cisplatin resistance in OS.

**Results:** We showed that GLI1 expression was up-regulated in OS patients and cisplatin-resistant cells. Silencing GLI1 significantly restored the sensitivity of OS to cisplatin, reduced proliferation, migration and cloning capacity of cisplatin sensitive and resistant cells, and increased the apoptosis rate *in vitro*. Furthermore, combined administration of GANT61 and cisplatin markedly inhibitted tumor growth in the mouse model. Mechanitic studies found that γ-H2AX is involved in the cisplatin resistance, and blockade of Hedgehog/GLI1 pathway increased the expression of γ-H2AX.

**Conclusion:** Abnormal activation of Hedgehog-GLI1 pathway can regulate the expression of γ-H2AX, thus affecting DNA damage and repair functions, and promoting acquired cisplatin resistance of OS.

## Introduction

Osteosarcoma (OS) is the most frequent primitive malignant bone malignancy originating from mesenchymal tissue. It is highly aggressive and occurs mainly in children and adolescents 1. The standard treatment for OS consists of neoadjuvant chemotherapy, including a combination of cisplatin (DDP), adriamycin and vincristine, followed by surgical resection and postoperative chemotherapy 2. While the application of multi-drug chemotherapy and improved surgical techniques have reduced the mortality of OS patients, rapid development of metastatic lesions and emergence of chemotherapy resistance greatly affected the therapeutic efficacy and survival rate of the patients. Therefore, it is important to develop improved treatment options and identify new therapeutic targets for reversing the chemoresistance in the treatment of OS.

Hedgehog signaling pathway is a key pathway for embryo formation and development, which controls both organ development and tissue homeostasis 3. Dysregulation of Hedgehog pathway is involved in various human diseases, including different types of cancer. Particularly, gene mutations in various components of Hedgehog signaling pathway are functionally linked to numerous tumors such as leukemia, basal cell carcinoma, and medulloblastoma 4-6. Hedgehog signaling acts on tumor cells, osteoclasts and stromal cells in a variety of host tumor microenvironments 7, and plays a regulatory role in the tumor microenvironments of oral squamous cell carcinoma 8 and prostate cancer 9. In addition, aberrant activation of Hedgehog pathway could lead to resistance to chemotherapy in tumor via distinct mechanisms 10.

Glioma-Associated Oncogene Homolog 1 (GLI1) is the key transcription factor of the Hedgehog pathway, while abnormal activation of GLI1 can activate the expression of multiple downstream targets, affecting tumor cell proliferation, apoptosis, DNA damage and repair (DDR), and other processes. GLI1 is closely related to the occurrence, development, invasion, metastasis, drug resistance, and prognosis of tumors 11, 12. Since the promotion of DDR protein expression through GLI1 overexpression and thus enhanced ability to repair DNA damage is one of the mechanisms of tumor resistance, while one of the mechanisms of DDP action is to cause DNA cross-linking and damage, the enhanced ability of repairing DNA damage caused by over-activated GLI1 is considered to be one of the reasons for DDP drug resistance 13. However, it remains unclear whether Hedgehog-GLI1 pathway plays an important role in DDP resistance in OS.

In this study, we performed *in vitro* and *in vivo* experiments to investigate the role of Hedgehog-GLI1 pathway in DDP resistance of OS as well as the underlying mechanism. This study could provide valuable data for evaluating whether Hedgehog pathway inhibitors can be used as chemotherapy sensitization agents for OS.

## Materials and methods

### Cell culture and transfection

Two human OS cell lines SOSP-9607 (Cisplatin sensitivity) and SOSP-9607/CR (Cisplatin Resistance) were recently established in our laboratory 14. The cells were grown in RPMI-1640 medium (Hyclone, USA) supplemented with 10% fetal bovine serum (Gibco, USA) in an incubator with 5% CO_2_ at 37 °C.

Small interfering RNA (siRNA) targeting GLI1 (siGLI1) and siRNA-NC were purchased from GenePharma (Shanghai, China). SOSP-9607 or SOSP-9607/CR cells were transfected with siGLI1 or siRNA-NC using Lipofectamine RNAiMAX (Thermo Fisher Scientific, USA) according to the manufacturer's instructions. The transfected cells were divided into two groups: SOSP-siGLI1 (SOSP/CR-siGLI1) and SOSP-NC (SOSP/CR-NC).

### Patient tissue samples

Eight patients with OS participated in this study. This study was conducted in accordance with the guidelines of the Declaration of Helsinki and was approved by the Ethical Committee of Tangdu Hospital of the Fourth Military Medical University (TDLL-2017151). The informed consent form was written and obtained from all participants. All patients underwent OS resection. None of the patients had undergone chemotherapy, radiotherapy, or any other treatment for cancer prior to surgery. Formalin-fixed paraffin-embedded OS samples were obtained from biopsies and matched excised tumors. The detailed clinical characteristics are in Table S1. The samples were sectioned as usual and used to assess GLI1 activity by Immunohistochemical (IHC).

### Cell viability

Cell viability was measured by the Cell Counting Kit-8 (CCK-8) assay. The cells were seeded into 96-well plates, incubated overnight and then treated with various concentrations of DDP (0-32 μM) for 24 hours. Thereafter, cells were incubated with 10 μl CCK-8 reagent (APExBIO, USA) for 2 hours, and the optical density (OD) at 450 nm was detected by a microplate reader.

### RNA isolation and qRT-PCR

Total RNA was extracted from the cells using RNAiso Plus (Takara, Japan) and reversely transcribed into cDNA using PrimeScript RT Master Mix (Takara, Japan). The cDNA was amplified using a TB Green Premix Ex TaqTM II (Tli RNaseH Plus) (Takara, Japan). The relative expression of GLI1 was calculated by the method of 2^-ΔΔCt^ and normalized to β-actin. The primers were as follows: GLI1: forward 5ʹ-TGAAACTGACTGCCGTTGGG-3ʹ and reverse 5ʹ-AGTATGACTTCCGGCACCCT-3ʹ; β-actin: forward 5ʹ-TTCCTTCCTGGGCATGGAGTCC-3ʹ and reverse 5ʹ- TGGCGTACAGGTCTTTGCGG -3ʹ.

### Protein extraction and western blot analysis

The protein was extracted from cells or tissues by using RIPA lysis buffer (Beyotime Biotechnology, Shanghai, China). The protein was separated by SDS-PAGE and then transferred to a PVDF membrane (Beyotime Biotechnology, Shanghai, China). After being blocked in 5% skim milk for 1 hour, the membrane was incubated with the primary antibody overnight at 4 °C, followed by an incubation with the secondary antibody for another 2 hours. Antibodies directed against GLI1, γ-H2AX and β-actin were purchased from Proteintech, USA. β-actin was included as an internal reference. Densitometry of the protein bands was performed using Image J package (Bio-Rad, USA).

### Cell apoptosis assay

Annexin V FITC Apoptosis Kit (BD Biosciences, USA) was utilized to measure the apoptosis rates. After digestion, resuspended samples were stained with Annexin V fluorescein isothiocyanate (FITC) and propidium iodide (PI). Cells were incubated for 30 minutes and then subjected to flow cytometry (BD Biosciences, USA).

### Wound healing assay

Cells in the logarithmic growth phase (2×10^5^/well) were seeded into 6-well plates. After 48 hours of culture, the cell monolayer was wounded using a plastic pipette tip. Thereafter, the cells were gently rinsed with PBS, and 1% FBS medium was added to the cells. The wound closure was observed, and representative images were photographed under a microscope at 0 and 24 hours, respectively.

### Colony formation assay

Cells were seeded in the culture plates and cultured (1.5×10^3^/well) in an incubator for 1 week at 37 °C in 5% CO_2_ atmosphere to form colonies. Plates were then stained with crystal violet solution for 30 minutes. Images were captured under a microscope, and the number of colonies was counted.

### Immunohistochemistry

IHC staining was performed on formalin-fixed and paraffin-embedded tissues or paraformaldehyde-fixed cells. After being treated with the blocking solution (0.1% Triton-X) for penetration, fixed tissues or cells were incubated with anti-GLI1 antibodies overnight at 4°C, followed by an incubation with the secondary antibody. Images were collected using a digital camera (Nikon, Japan).

### Tumor xenograft assay

The animal experiment was conducted in accordance with the Guidelines for the Care and Use of Laboratory Animals of the National Institute of Health in China and approved by the Ethical Committee of Tangdu Hospital of the Fourth Military Medical University (TDLL-2017151). SOSP-9607/CR cells were injected into BALB/c nude mice (6 weeks old) to generate the animal model. The mice were then randomly divided into three groups: Ctrl group; DDP group; and GANT61+DDP group. Seven days after the injection, tumor volume was measured every 7 days and calculated using the formula: (length × width^2^)/2. Tumor weights were determined 28 days after the injection.

### Statistical analysis

Statistical analysis was performed by GraphPad Prism v8.0.2. All data are presented as means ± standard deviation. Student's t-test or one-way analysis of variance was conducted to assess the statistical significance of differences among groups. A p<0.05 value was considered significant.

## Results

### High expression of GLI1 in OS patients and drug-resistant cells

**Figure 1:**
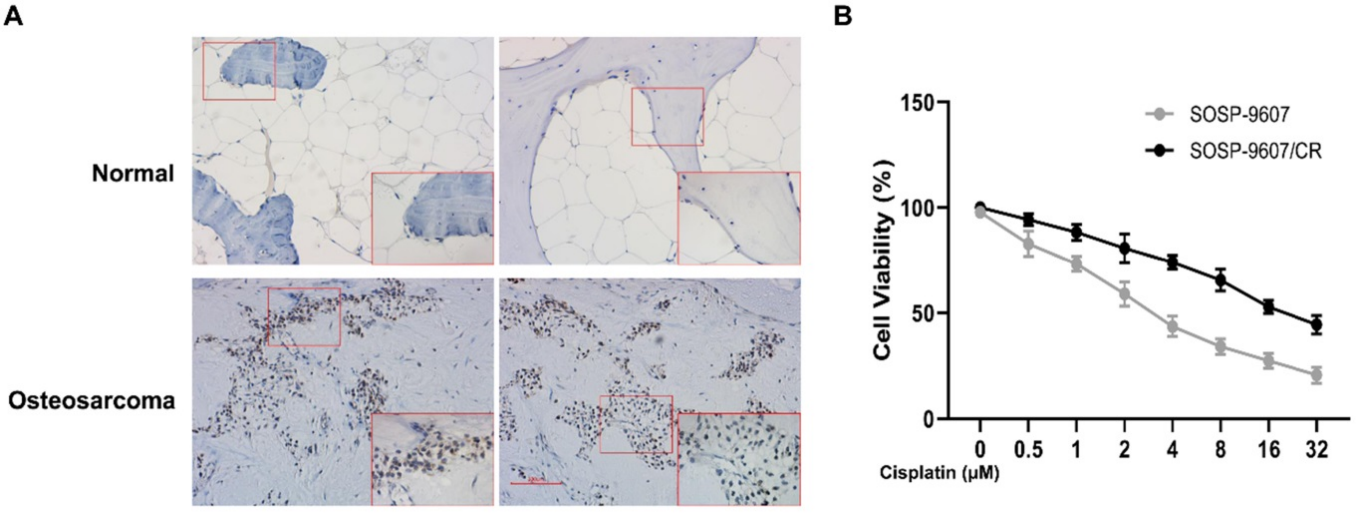
** Expression of GLI1 in patient samples. (A)** Immunohistochemistry of OS tumor tissues and normal bone tissues (magnification 100x). **(B)** CCK8 assay-based detection of the IC50 of DDP in SOSP-9607 and SOSP-9607/CR cells. *p<0.05.

**Figure 2:**
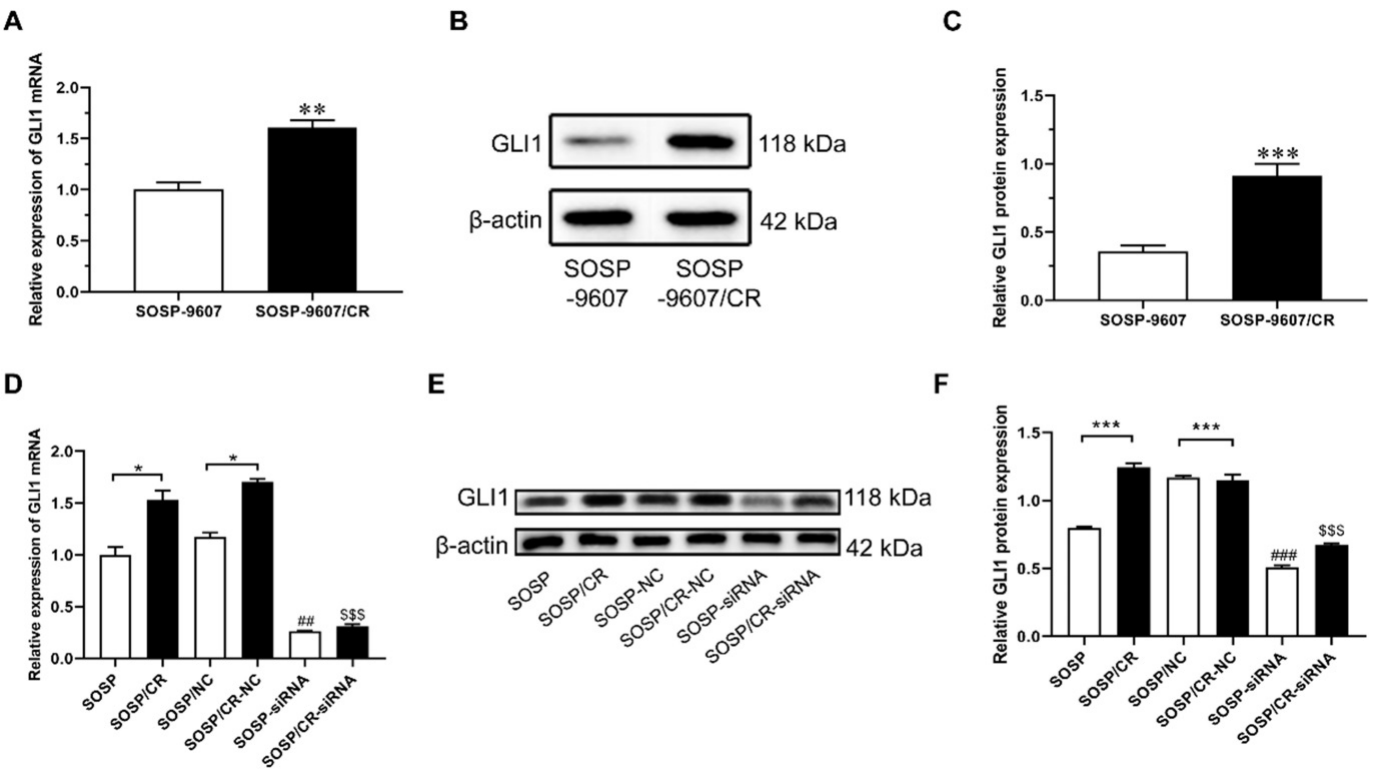
** Expression levels of GLI1 in SOSP-9607 and SOSP-9607/CR cells.** The mRNA (**A**) and protein (**B-C**) expression levels of GLI1 in SOSP-9607 and SOSP-9607/CR cells were detected by qRT-PCR and western blot analysis. ^**^p<0.01, vs. the SOSP-9607 group, ^***^p<0.001;^ ##^p<0.01, vs. the SOSP group, ^###^ p<0.001; ^$$^p<0.01, vs. the SOSP group, ^$$$^p<0.001.

To investigate the role of GLI1 in human OS, we performed IHC to evaluate the expression of GLI1 in the specimens of OS patients. As illustrated in Figure 1A, a higher expression level of GLI1 was detected in OS tumor tissues as compared to the normal bone tissues. To determine the role of GLI1 in the drug-resistant cells, we first constructed a drug-resistant cell line and verified its sensitivity to DDP using the CCK-8 assay. The CCK-8 assay showed that the IC50 values of DDP in cell lines SOSP-9607 and SOSP-9607/CR were 3.81 μM and 20.89 μM, respectively (Figure 1B). These data indicated that SOSP-9607 cells were sensitive to DDP, while SOSP-9607/CR cells were resistant to DDP. The qRT-PCR assay and western blot analysis further revealed that SOSP-9607/CR cells displayed a significantly higher expression level of GLI1 than SOSP-9607 cells (Figure 2A-C). Collectively, these findings suggested that the overexpression of GLI1 in OS tumor tissues and cisplatin-resistant cells may be functionally linked to osteosarcoma progression.

### Knockdown of GLI1 increases cell apoptosis while decreasing cell proliferation, colony formation and migration

**Figure 3:**
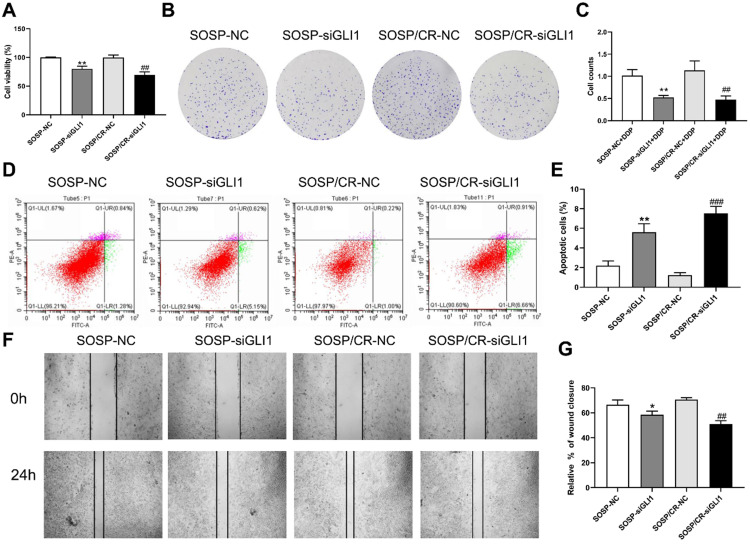
** Effect of silencing GLI1 on the biological behavior of OS cells. (A)** CCK-8 assays were performed to evaluate cell proliferation. **(B, C)** Colony formation assay was performed to detect the clone formation capacity. **(D, E)** Apoptosis of SOSP-9607 and SOSP-9607/CR cells was detected by flow cytometry. **(F, G)** Scratch area healing in each group after scratched for 0 and 24h (100×). ^*^p<0.01 vs. the SOSP-NC group, ^**^p<0.01; ^##^p<0.01 vs. the SOSP/CR-NC group, ^###^p<0.001.

To further examine the effects of Hedgehog-GLI1 pathway on biological behaviors of OS cells, we analyzed the proliferation, colony formation, apoptosis, and migration of SOSP-9607 and SOSP-9607/CR cells with down-regulated expression of GLI1. As shown in Figure 2D-F, GLI1 expression was significantly decreased in SOSP-9607 and SOSP-9607/CR cells treated with GLI1 siRNA. Notably, while silencing GLI1 promoted apoptosis and decreased proliferation, colony formation and migration in the two cell lines (Figure 3A), this effect was more pronounced in SOSP-9607/CR cells (Figure 3B-G). These observations indicated an inhibitory effect of GLI1 downregulation on growth and metastasis of OS cells.

**Figure 4:**
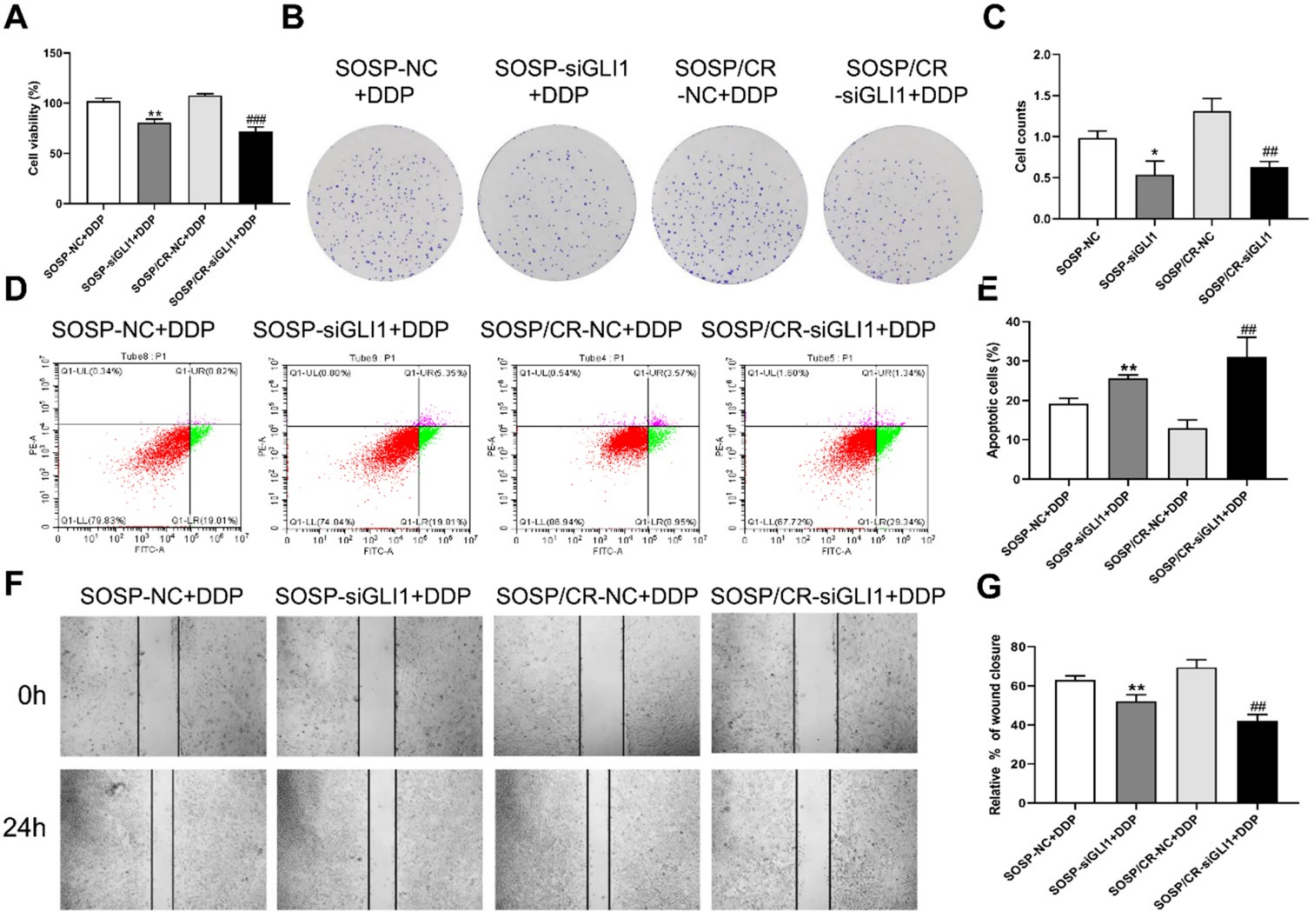
** Effect of GLI1 on the biological behavior of OS cells after the addition of DDP.** After 80 μM DDP addition (**A**) CCK-8 assays were performed to evaluate cell proliferation. **(B, C)** Colony formation assay was performed to detect the clone formation capacity. **(D, E)** Apoptosis of SOSP-9607 and SOSP-9607/CR cells was detected by flow cytometry. **(F, G)** Scratch area healing in each group after scratched for 0 and 24h (100×). ^*^p<0.01 vs. the SOSP-NC group, ^**^p<0.01; ^##^p<0.01 vs. the SOSP/CR-NC group, ^###^p<0.001.

We next investigated the sensitivity of the above cells to DDP. As shown in Figure 4, DDP treatment (80 μM) led to a further increase in the apoptosis rate as well as a further decrease in proliferation, colony formation and migration in the two cell lines with reduced expression of GLI1, indicating that silencing GLI1 enhances DDP-mediated anti-proliferative effects on the OS cells.

### DNA damage protein γ-H2AX regulates cell resistance

**Figure 5:**
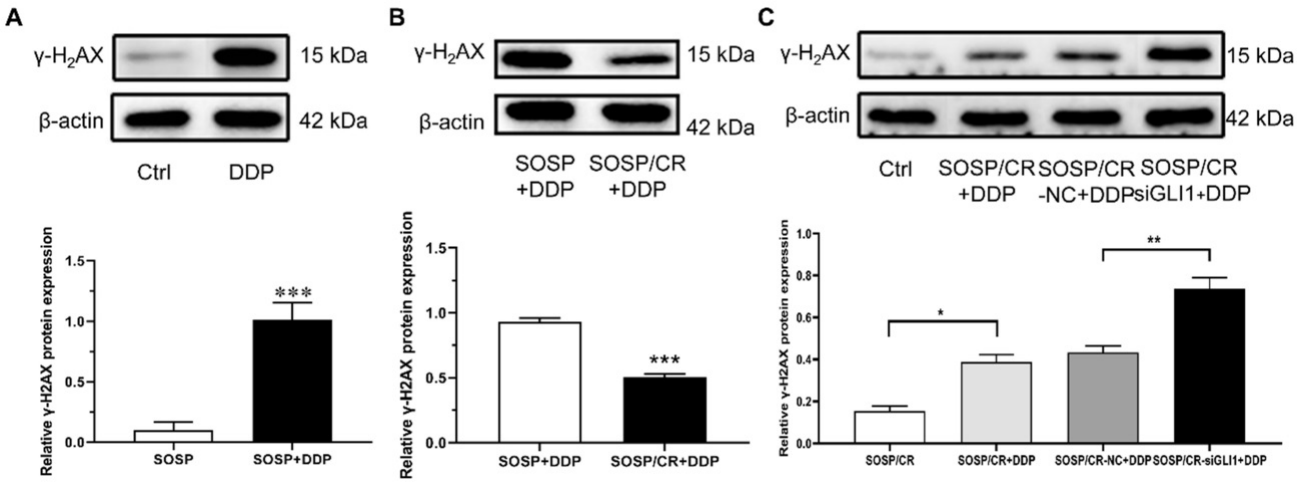
** Mechanisms of GLI1-associated DDP resistance on OS cells.** The expression of γ-H2AX in SOSP+DDP group (**A**), SOSP/CR+DDP group (**B**) and SOSP/CR+siGLI1+DDP group (**C**) was detected by western blot analysis. ^*^p<0.05, ^**^p<0.01, ^***^p<0.001.

Given that γ-H2AX plays a role in the retention of repair at sites of DNA damage 15, we chose to analyze the expression of γ-H2AX to explore the mechanism of GLI1-related DDP resistance. For this purpose, we treated the cells with DDP and examined the expression of γ-H2AX. As depicted in Figure 5A and B, while DDP treatment markedly increased γ-H2AX expression in SOSP-9607 cells, a significantly lower level of γ-H2AX was evident in SOSP-9607/CR cells compared with SOSP-9607 cells following the DDP treatment. To determine whether silencing GLI1 enhances DDP-induced DNA damage, the protein levels of γ-H2AX were measured in SOSP-9607/CR cells. As shown in Figure 5C, γ-H2AX expression was significantly increased in cells treated with DDP and siGLI1 as compared to those treated with DDP alone. Together, these data suggested that GLI1 downregulation suppresses the repair of DDP-mediated DNA damage in SOSP-9607/CR cells.

### Hedgehog-GLI1 pathway regulates drug resistance *in vivo*

**Figure 6:**
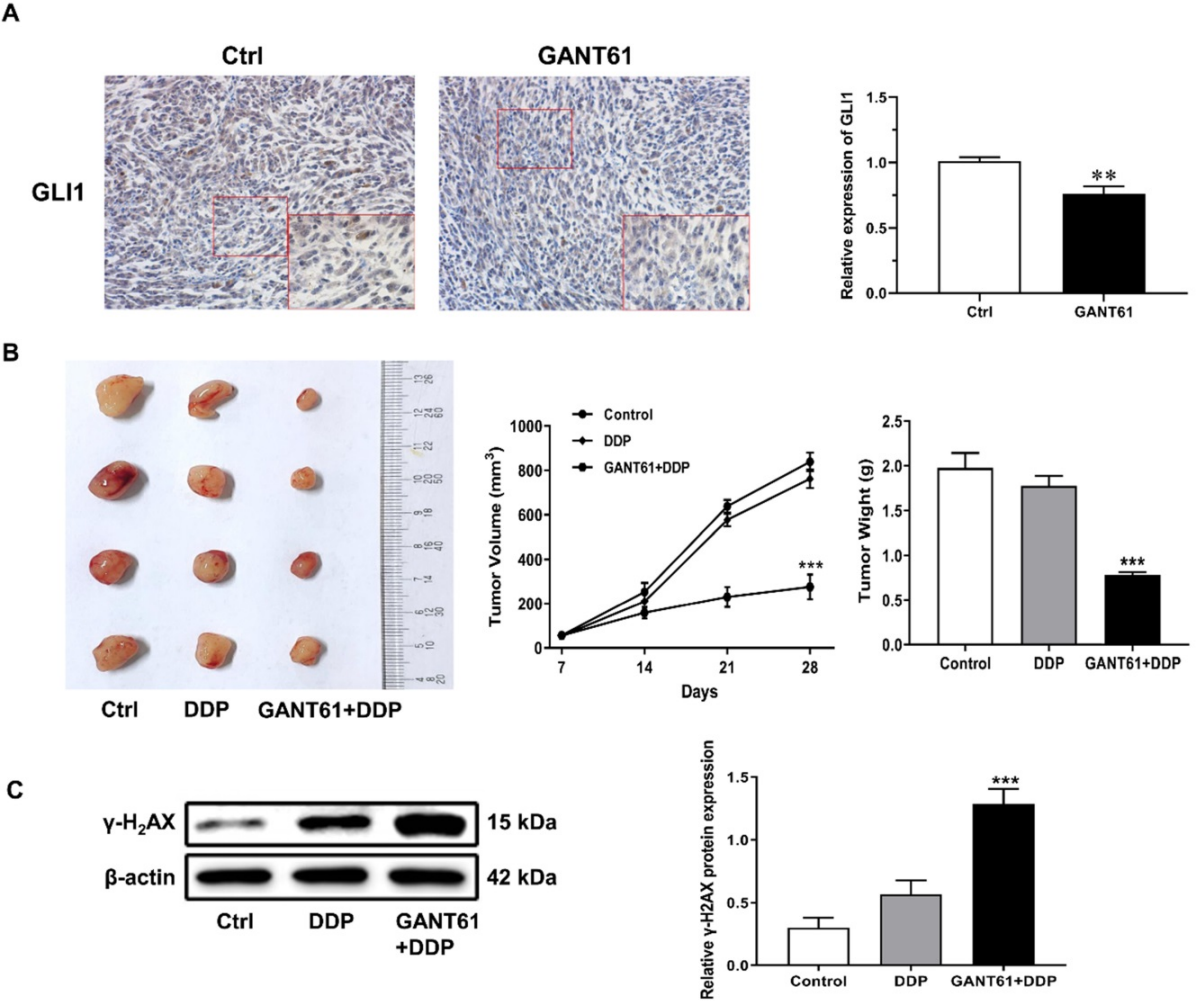
** Role of the Hedgehog-GLI1 pathway in the *in vivo* resistance of OS to DDP. (A)** IHC was performed to detect the expression level of GLI1 in the mouse model treated with GANT61.^ **^P<0.01. **(B)** The tumor volume and weight of the mice treated as indicated were measured and analyzed. **(C)** The protein levels of γ-H2AX in xenograft tumors were assayed using western blot analysis. ^***^P<0.001 vs. the DDP group.

To further determine whether Hedgehog-GLI1 pathway plays a role in OS resistance to DDP *in vivo*, a subcutaneous implant nude mouse model was generated by using SOSP-9607/CR cells and treated with Hedgehog pathway inhibitor drug GANT61 combined with cisplatin (Figure 6A). As shown in Figure 6B and 6C, xenografts of SOSP-9607/CR cells in mice treated with DDP and GANT61 were significantly more sensitivity to DDP than those in mice treated with DDP alone, while co-treatment with GANT61 and DDP induced a significantly higher expression of γ-H2AX than DDP only. Overall, these results demonstrated that GANT61 can increase sensitivity of the cells to DDP presumably by up-regulating the expression of γ-H2AX *in vivo*.

## Discussion

While Hedgehog-GLI1 pathway regulates tumor resistance via distinct mechanisms 16, 17, it remains unclear whether the pathway plays an important role in the resistance of OS. Here, we comprehensively employed molecular and cellular biology techniques to investigate the role of Hedgehog-GLI1 pathway in regulating DDP resistance in OS as well as the underlying molecular mechanism. The findings in this study may contribute to the identification of potential targets for the development of new drugs to reverse the resistance of OS.

GLI1 is one of the key components of Hedgehog pathway, while its dysregulation could lead to chemoresistance in many malignant tumors including colorectal cancer 18, gastric cancer 19, lung cancer 20, and glioblastoma 21. As a transcription factor, GLI1 can change tumor chemoresistance via epigenetic regulation of some chemotherapy targets. For examples, GLI1 can bind to the promoter region of O6-methylguanine DNA methyltransferase (MGMT) gene to regulate the expression of MGMT and chemotherapy resistance to temozolomide in glioblastoma cells 21. Likewise, in gastric cancer, GLI1 protein was found to up-regulate the expression of ATP-binding cassette sub-family G member 2 (ABCG2) via the interaction between Gli-binding consensus site and promoter fragment of ABCG2, thus changing cytochemical resistance 22. To determine the role of GLI1 in DDP resistance in OS, we firstly detected the expression of GLI1 in resistant cells and found that the expression of GLI1 in SOSP-9607/CR cells was significantly higher than that in SOSP-9607 cells. Then, we showed that down-regulation of GLI1 sensitized DDP-induced cytotoxicity *in vitro*, indicating a correlation between GLI1 and DDP resistance. Moreover, combined administration of GLI1 inhibitor and DDP led to impaired tumor growth in the mouse model. Taken together, we concluded that Hedgehog-GLI1 pathway plays an important role in DDP resistance in OS.

Multiple studies have reported a link between DDR and DDP resistance. The PI3K/AKT/mTOR signaling pathway is an essential mediator of cell growth, survival and motility and is associated with increased cisplatin resistance in OS cells 23. Studies have shown that down-regulation of Microtubule Affinity Regulated Kinase 2 (MARK2) can inhibit the activity of PI3K/Akt/mTOR pathway, leading to a decrease in the expression of DNA-dependent protein kinase, a primary indicator of DDR, and DDP resistance of OS stem cells 24. In addition, it was reported that HOXB7 interacted with Ku70, Ku80 and DNA-PKcs to induce cell cycle arrest. Downregulation of HOXB7 expression or disrupt its function enhanced DDP sensitivity in esophageal squamous cell carcinoma 25. Based on these observations, we further investigated the mechanism underlying regulatory role of Hedgehog-GLI1 pathway in DDP resistance by focusing on DDR. To this end, we examined whether the activation of Hedgehog-GLI1 pathway enhances DDR and causes DDP resistance. In the experiments, we analyzed the expression of the γ-H2AX, a marker of DNA damage, to assess the degree of DNA damage. The analysis revealed that while DDP could cause DNA damage in SOSP-9607 cells, a significantly lower level of DNA damage was detected in SOSP-9607/CR as compared to SOSP-9607 cells. Strikingly, inhibition of Hedgehog GLI1 signaling can restore DDP-caused DNA damage in SOSP-9607/CR cells to a certain extent both *in vitro* and *in vivo*. Overall, these data suggest that activated Hedgehog-GLI1 pathway can reduce DNA damage presumably by down-regulating γ-H2AX, thereby enhancing DDP resistance.

**Figure 7:**
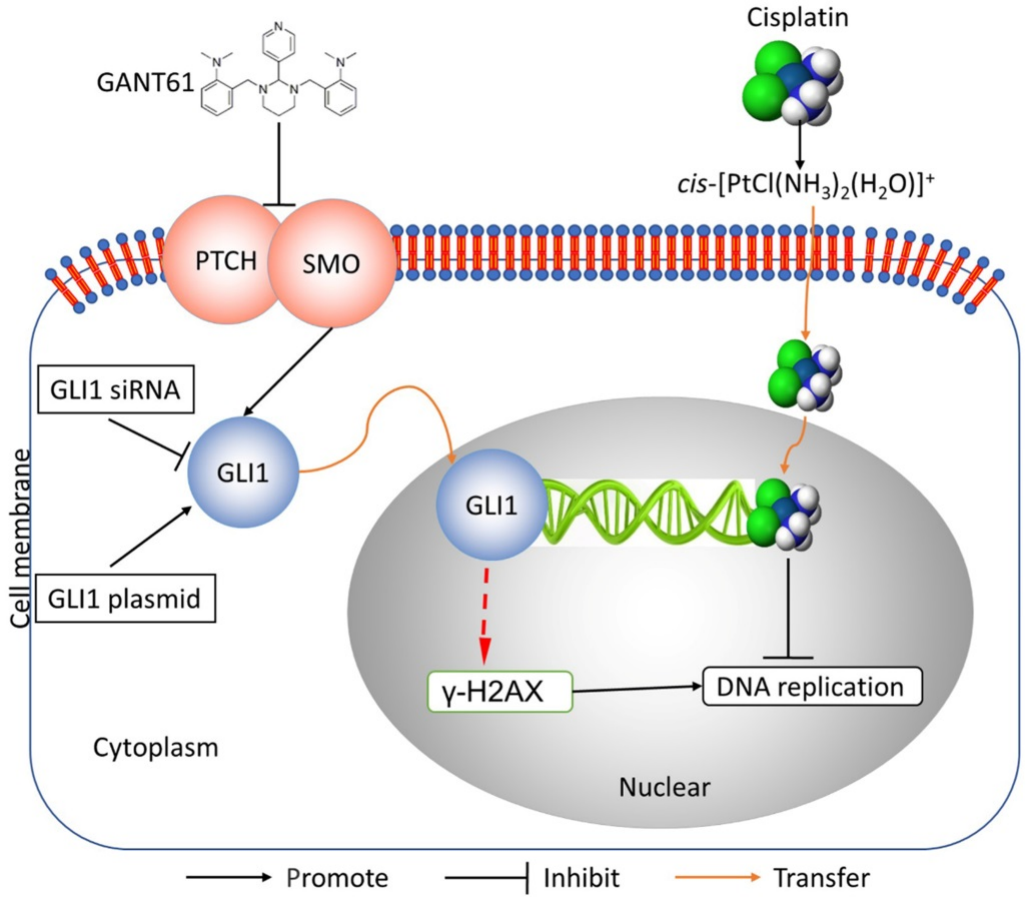
Schematic diagram of potential mechanisms underlying GLI1 regulation of the resistance to cisplatin in human osteosarcoma.

In this study, we showed the effects of Hedgehog GLI1 pathway on OS resistance *in vitro* and *in vivo*. Furthermore, we found that highly expressed GLI1 regulates the sensitivity of OS to DDP presumably by inhibiting the expression of γ-H2AX (Figure 7). The findings suggest that combined administration of the Hedgehog signaling inhibitor and DDP may be a promising strategy to improve drug-resistant in patients with OS.

## Supplementary Material

Supplementary table.
