# Supplementary material for: Hedgehog/GLI1 signaling pathway regulates the resistance to cisplatin in human osteosarcoma
Source: J Cancer. 2021 Sep 21;12(22):6676–84. doi: 10.7150/jca.61591 (PMC8518013; doi:10.7150/jca.61591)
Supplement: Supplementary file 1 — Supplementary table. [file jcav12p6676s1.pdf]

**Supplementary Table 1. Clinical information of samples from patients with osteosarcoma**

| Number(n=8) | Age (year) | Gender | Tumor type | Clinical stages |
|---|---|---|---|---|
| 1 | 84 | female | Osteoblastic osteosarcoma | - |
| 2 | 9 | female | Common osteosarcoma | IIB |
| 3 | 17 | male | Common osteosarcoma | - |
| 4 | 20 | male | Fibroblastic osteosarcoma | IIB |
| 5 | 13 | male | osteosarcoma (with necrosis after chemotherapy) | III |
| 6 | 17 | male | Common osteosarcoma | IIB |
| 7 | 18 | male | Common osteosarcoma | IIB |
| 8 | 15 | male | Multiple/metastatic osteosarcoma | - |
